# The role of domestication and experience in ‘looking back’ towards humans in an unsolvable task

**DOI:** 10.1038/srep46636

**Published:** 2017-04-19

**Authors:** Sarah Marshall-Pescini, Akshay Rao, Zsófia Virányi, Friederike Range

**Affiliations:** 1 Comparative Cognition, Messerli Research Institute, University of Veterinary Medicine, Vienna, Medical University of Vienna, University of Vienna, Veterinaerplatz 1, 1210 Vienna, Austria; 2 Wolf Science Centre, Dörfles 48, 2115 Ernstbrunn, Austria

## Abstract

A key element thought to have changed during domestication is dogs’ propensity to communicate with humans, particularly their inclination to gaze at them. A classic test to measure this is the ‘unsolvable task’, where after repeated successes in obtaining a reward by object-manipulation, the animal is confronted with an unsolvable version of the task. ‘Looking back’ at humans has been considered an expression of dogs seeking help. While it occurs more in dogs than in socialized wolves, the level of exposure to human communication also appears to play a role. We tested similarly raised adult wolves and mixed breed dogs, pet dogs and free-ranging dogs. Unlike previous studies, as well as species and levels of socialization, we included ‘persistence’ in trying to solve the task as a potential explanatory factor. Wolves were more persistent than all dog groups. Regardless of socialization or species, less persistent animals looked back sooner and longer. Free-ranging dogs, despite little exposure to dog-human communication, behaved similarly to other dogs. Together, results suggest that basic wolf-dog differences in motivation and exploration may override differences in human-directed behaviour when animals are equally socialized, and that once the human is considered a social partner, looking behaviour occurs easily.

Domestication is thought to have changed dogs’ ability to communicate and cooperate with humans^1^,^2^,^3^,^4^. One key element of this communication is ‘looking back’ towards a human when confronted with an unsolvable task; this behaviour is considered to be a communicative act aimed at seeking human assistance. Indeed, in early studies comparing wolves and malamutes, Frank & Frank (1985) presented both with puzzle boxes of increasing complexity and noted how wolf pups would ‘attack each puzzle immediately’ and persist ‘until the problem was solved or time ran out’ (pp. 271) in contrast to dog pups who quickly reverted to seeking human attention upon discovering that the food was not immediately available. In a seminal study comparing wolves and dogs, Miklósi and colleagues found that when confronted with an unexpectedly unsolvable task, 4 month-old pet dogs were more likely to “look back” to their owner and did so sooner and for longer than wolves of the same age raised in a ‘pet-like’ environment^3^. Since this first study, the propensity to look back in dogs has been shown to differ between breed-groups^6^,^7^ and to have a genetic basis^8^,^9^, providing further circumstantial support for a potential effect of domestication on this behaviour.

Additionally, a variety of studies using the same paradigm have also shown that dogs’ experience with dog-human communication may affect their looking back behaviour. For example, kennelled dogs (Labrador retrievers) with reduced exposure to humans from birth (i.e. limited to daily contact with humans cleaning their kennels and putting down a bowl of food) show a higher latency and shorter duration of looking back in an unsolvable task paradigm compared to breed-matched pet dogs^10^. Conversely, dogs that engage in activities which require constant intensive coordination with their owners (e.g. agility) show a higher propensity to look back in such tasks than pet dogs with no specific training experiences and dogs trained for more independent tasks (i.e. search and rescue)^11^. Furthermore, in two large-scale studies (175 and 125 dogs tested respectively) using the unsolvable task with a non-trained pet dog population, older dogs spent longer looking back than younger animals, leading authors to conclude this was likely an effect linked to their longer experience with humans in comparable situations^6^,^7^. Taken together, these results highlight that both the degree and type of interaction with humans has strong effects on their ‘looking back’ behaviours in such tasks.

To further elucidate the roles of domestication and experience with human interactions in the ‘looking back’ response, we used the unsolvable task paradigm to test adult wolves (N = 15) and mixed-breed dogs (WSCD N = 14) at the Wolf Science Center that, having being raised and kept in the same manner from birth, have been equally exposed to human communication (see Table S1 in Supplementary Material). Furthermore, we tested two populations of dogs with very different experiences of dog-human communication i.e. adult mixed breed pet dogs living in Vienna, Austria (PD N = 19) and free-ranging dogs in India (FRD N = 11, see Fig. 1). The free-ranging dogs tested lived on the streets, were mostly dependent on scavenging on human refuse and although friendly with humans, had no known established relationship with any specific person/s. Based on this, we considered them representative of a more independent dog population with noticeably less experience of humans helping them to obtain out-of-reach objects/food compared to pet dogs living in close contact with their owners in a Western, urbanized environment.

Similar to previous studies, the task consisted of three trials in which subjects could overturn a container to obtain food, followed by a single trial in which the container was fixed to a board, thereby making the task unsolvable^11^. We analysed data comparing the groups on their likelihood and latency to look back, the duration and frequency of gazing at a human, and gaze alternation behaviours (i.e. the frequency of looking at the apparatus and then a human or vice versa). Furthermore, since a number of studies using different problem solving tasks have shown that wolves are more persistent than dogs in such tasks^5^,^12^ and more persistent animals are also those that look back less frequently^13^,^14^,^15^, unlike previous studies using the ‘unsolvable paradigm’, we included persistence (i.e. the time spent interacting with the apparatus) as a potential explanatory factor alongside group (i.e. wolves, equally raised dogs, pet dogs and free-ranging dogs) in all our analyses (see ‘Analyses’ section below). We also extended the unsolvable trial to 3 minutes (compared to most studies in which animals had just 1 or 2 minutes to attempt the task^3^,^6^,^7^) to potentially allow more persistent animals to also exhibit ‘looking back’ behaviours. We nevertheless also report results of group comparisons when persistence was not factored into the analyses to allow for greater comparability with previous studies.

## Results

Results showed that across *solvable trials*, animals in all groups improved significantly in the latency to obtain the reward (mean: Trial 1 = 19.5; Trial 2 = 6.6; Trial 3 = 4.9 seconds; LMM: χ^2^ = 11.72, p < 0.001). However, in all trials, wolves were faster at accessing the food than all dog groups (LMM: χ^2^ = 53.9, p < 0.001; mean: wolves 4 seconds vs. WSCD 12.2 seconds χ^2^ = 5.1, vs. PD 6.4 seconds χ^2^ = 5.6, vs. FRD 20.8 seconds χ^2^ = 7.7, all p < 0.001) and free-ranging dogs tended to be significantly slower than pets (χ^2^ = 2.5, p = 0.057) (see Tables S2 and S3 in Supplementary Materials for full results).

**Figure 2:**
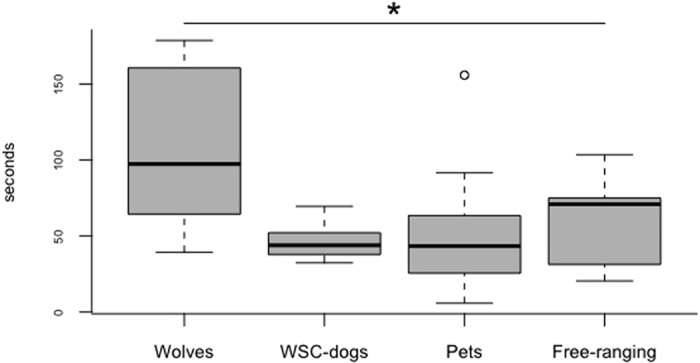
Time (in seconds) spent interacting with the apparatus during the unsolvable trial by animals in the different groups (mean, interquartile range and upper-lower limits).

In the *unsolvable trial*, while wolves spent more time interacting with the apparatus than all dog groups, dog groups did not differ from one another (LM: df = 3, F = 9.08, p < 0.0001; wolves vs. WSCD t = 4.32; wolves vs. PD t = 4.73 both p < 0.001; wolves, vs. FRD t = 3.12, p = 0.015) (Fig. 2 and Table S4 in Supplemental Material). These results support studies showing that wolves are more persistent than dogs in manipulative tasks^5^,^12^.

In the unsolvable trial, 11/15 wolves, all 14 WSC dogs, all 19 pets and all 11 free-ranging dogs looked back towards a human. The group difference was significant (GLM: χ^2^ = 11.8, p = 0.008) and persistence (χ^2^ = 21.7, p < 0.001) also significantly affected the likelihood of looking back (Table S5 Supplementary Material). Interestingly, 3 of the 4 wolves that did not look back at all spent more than 170 seconds (94% of the total trial duration) interacting with the apparatus. Accordingly, it is possible that had we extended the testing time further, these animals would have eventually looked towards a person as well.

**Figure 3:**
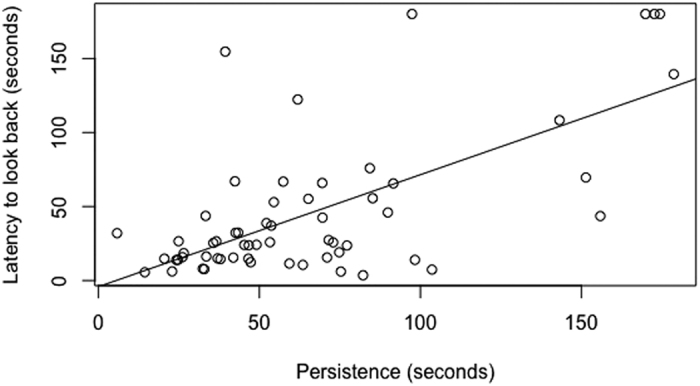
Linear positive relationship between persistence and the latency to look back at the person. The more animals spent interacting with the apparatus the longer they took to look back at the person.

Considering only the animals that looked at a human in the unsolvable trial (wolves N = 11, WSCD N = 14; PD N = 19; FRD N = 11): no group difference emerged in the latency to look back (Table S6 Supplementary Material); rather, regardless of group, the longer an individual spent interacting with the apparatus, the longer it took for them to look back at a person (LM: F = 11.9, p = 0.001) (Fig. 3). No group difference emerged (LM: F = 2.62, p = 0.06), even when persistence was not considered. Furthermore, no group difference emerged in the duration of looking back; rather, regardless of group, the more time animals spent interacting with the apparatus the less time they spent looking at a person (LM: F = 33.4, p < 0.001; Table S7 Supplementary Material). Analogous results emerged for the frequency of looking back: regardless of group, the more time an animal spent interacting with the apparatus the less frequently it looked back (GLM: χ^2^ = 11.39, p < 0.001: Table S8 Supplementary Material). When *not* including persistence as an explanatory factor, a group effect emerged on the duration of looking (LM: F = 8.4, p < 0.001) with wolves looking towards humans for shorter periods than dogs in all groups, although no difference emerged amongst the latter (Table S9 Supplementary Material). Similarly, when not including persistence, a group effect emerged on the frequency of looking back behaviour GLM: χ^2^ = 16.09, p = 0.001), with wolves looking towards humans less often than dogs in all groups, although no difference emerged amongst the latter (Table S10 Supplementary Material).

Finally, for frequency of ‘gaze alternations’ (i.e. looks to the apparatus immediately followed or preceded by a look to a person), a behaviour that has been considered a more stringent measure of communicative behaviour between dogs and humans in such tasks^16^, a group effect emerged (GLM: χ^2^ = 8.75, p = 0.034; Table S11 Supplementary Material). However, corrected post-hoc comparisons showed only a marginally significant difference between pet and WSC dogs (z = 2.55, p = 0.05; Table S12 Supplementary Material). Regardless of group, the more time spent interacting with the apparatus, the fewer gaze alternation behaviours were exhibited (GLM: χ^2^ = 11.39, p < 0.001; Table S11 Supplementary Material).

## Discussion

Overall, these results highlight the strong link between persistence in attempting to solve the task and different measures of looking back towards humans. In fact, when persistence is factored into the analyses, group differences in human-directed gazing behaviours do not emerge in our study populations but rather, regardless of group, the less persistent an animal, the sooner, longer and more frequently will it look back.

Since the current study was the first to include persistence as a potential explanatory factor (but see ref. 12 for a similar suggestion), we also ran analyses without this variable to allow us to compare results to previous studies using this paradigm. Indeed, when not including persistence in the analyses, the results replicate (with adults) the wolf-dog differences shown in 4-month old juveniles by Miklósi *et al*.^3^. However, when taking persistence into account, this emerged as the better explanatory variable in our sample, indicating that in this task, species differences occur rather in the persistence tendencies of dogs and wolves than in their readiness to look at humans (as suggested by ref. 12).

One interesting possibility is that wolves, with their stronger physical capabilities, may have a different perception of was is ‘unsolvable’ than dogs, and hence show more tenacity in their attempt to obtain the hidden food reward. We partially took this into account by presenting wolves with a stainless steel apparatus, still, this possibility cannot be completely excluded. Nevertheless it is interesting to note that not only have wolves been shown to be more persistent than pet dogs in manipulative tasks involving food^12^, but they have also been shown to be more explorative than similarly raised dogs both as adults^17^ and as pups^18^, even when when confronted with novel objects and environments with no food involved. Together, these results raise the hypothesis that more basic wolf-dog differences linked to their explorative and independent problem solving behaviours may have ‘knock-on’ effects on their interaction with people in such tests. Indeed, in studies in which human-directed gazing behaviour is measured without the potentially confounding variable of persistence, for example in a ‘showing’ task where animals need to indicate the location of hidden/unavailable food to an ‘ignorant’ human, equally raised wolves and dogs show the same capacity to communicate with their human partner^19^. Further support also comes from studies showing that wolves, when exposed from puppyhood to similar experiences as dogs, are equally, if not better, at following human gazing cues into distant space and around barriers^20^,^21^ and do not differ in their capacity to learn from human partners^22^.

These results suggest that, while dogs may have a genetic predisposition enabling them to form close relationships with humans with relatively little exposure^23^, when wolves are intensively socialized their communication with humans resembles that of similar socialized and kept pack dogs. A further question is whether with even more intensive (e.g. pet-like) socialization wolves would equal the performance of pet dogs that, in some studies, show even more sophisticated communicative interactions with humans than pack dogs^19^. Nevertheless, current results suggest that gazing behaviours towards humans are not necessarily a direct effect of domestication^24^, but potentially a behaviour that emerges as a result of animals’ acceptance of humans as social partners^25^.

Interestingly, very few behavioural differences emerged between the studied dog populations. While free-ranging dogs were slightly slower at solving the task in the ‘solvable’ trials than other dog groups - perhaps due to their limited experience with these kinds of objects and situations - looking back did not vary across groups. This is surprising to some extent, since if ‘looking back’ is to be interpreted as a communicative act by which a dog ‘looks for help’ from its human partner, we would expect dogs with a vastly greater experience of humans helping them (i.e. pet dogs) to be much more inclined to exhibit such a behaviour compared to free-ranging dogs living as independent scavengers.

So why did our study populations, particularly pet and free-ranging dogs, show such similar behavioural patterns in the current task? The most likely possibility is that populations were more homogenous than expected in the crucial aspects affecting looking back, i.e. in their persistence on the task and their level of socialization to humans as social companions and potential food-providers. Indeed, in terms of persistence, there was no difference between groups, with mean time spent interacting with the apparatus ranging between 46 and 61 seconds out of the 3 minutes provided. This appears quite comparable overall to Passalacqua *et al*.^6^ and Konno *et al*.^7^ who both found the mean duration of interaction to be approx. 30 seconds with a test duration of 1 minute. Furthermore, dog groups did not differ in the time spent interacting with the human during testing (see Table S13 Supplementary Material), which provides some evidence that they showed equal levels of ‘friendliness’ towards people in this kind of situation. It has been suggested that the ability to gain ‘human favour’ may be a crucial element affecting survival of free-ranging dogs^26^, and looking at people may be a crucial behaviour to obtain such ‘favour’. It is thus possible that the natural socio-ecology of the free-ranging dogs we tested (in terms of their reliance on human refuse and hand-outs, and early exposure to the human social environment) provided the necessary and sufficient conditions for them to show similar levels of persistence and social behaviour towards humans as the other dog populations tested.

In sum, ‘looking back behaviour’ is strongly linked to when an animal gives up trying to solve the unsolvable task. While we found no effect of the dogs’ prior experience to humans helping them, more persistent dogs were less inclined to look back towards a human. Furthermore, wolves were more persistent in the task than dogs and this largely explains why they took longer and looked less frequently and for a shorter time towards humans than dogs. Given the confounding effect of persistence on looking behaviour in the unsolvable task, future studies should aim at designing tasks allowing an independent assessment of these two variables and a better understanding of the causal link between them.

## Methods

### Subjects

#### Similarly raised and kept wolves and dogs

15 wolves (3F, 12M; mean age in years: 1.89, range: 1.09 to 3.7) and 14 mixed-breed dogs (4F, 10M: mean age in years: 1.02, range: 0.96 to 1.06) housed at the Wolf Science Center were tested. Wolves and dogs at the WSC (www.wolfscience.at) are raised and kept in the same way, and participate in various behavioural tests every week where they are rewarded with food. All wolves and dogs live in conspecific packs, but are worked in separation from their pack members daily. Participation in all training and testing sessions is voluntary. For more details relating to the upbringing and keeping of the animals please see Range & Virányi (ref. 20) and Supplemental Material.

#### Free-ranging dogs

Free-ranging dogs were approached on the streets of Mumbai, India and on the campus of the Indian Institute of Science, Bangalore, India. Dogs that visually appeared to be over 2 years of age and appeared solitary when spotted were preferentially chosen to avoid interference from other individuals. A pre-test (see Supplemental Material) was conducted to assess whether the dog was willing/comfortable enough to participate in the experiment. The pre-test was carried out with 46 dogs, 16 of which did not eat the food or were too wary to approach. A total of 31 free-ranging dogs proceeded to be tested but 8 dogs were not able to solve the task even after the experimenter attempted to demonstrate a solution and were hence not tested with the unsolvable version of the task. 6 dogs were excluded from analysis because other dogs approached and interfered with the procedure during testing, 1 dog was excluded because a human interfered during testing, 1 dog was excluded because he had an injury on his leg, and 3 dogs, although completing the test, succeeded in breaking the apparatus during the ‘unsolvable’ task, thereby curtailing the duration of the test. Hence, a total of 11 free ranging dogs (1F, 10M) were included in the analyses.

#### Pet dogs

Mixed-breed pet dogs were tested in two dog parks in Vienna. Dog owners were approached and asked whether they would like their dog to participate in a “cognition task” aimed at comparing different populations of dogs to wolves. Owners were asked about their dogs’ age, sex and whether the dogs had previously participated in any cognitive tasks. Only mixed-breed dogs over 1.5 years of age with no prior experience with cognitive testing and no high-level training experience were used to match the group of free-ranging dogs as much as possible. A total of 25 pet dogs were tested, however when testing 6 of these, other dogs in the park approached and interfered with the procedure, hence these dogs were excluded from analyses. The final sample consisted of 19 mixed breed pet dogs (10F, 9M mean age: 7 years; range: 3 to 12 years).

### Apparatus

A food reward (meat for wolves and WSC dogs, and pieces of sausage for pets and free-ranging dogs) was placed on a wooden board (approximately 60 cm × 30 cm) and covered with an overturned container (a commercial Tupperware container measuring approximately 15 cm^3^ for the dogs and a stainless-steel bowl measuring 30 cm in diameter for wolves). The containers had holes punched into them to allow the animals to smell the food. In the unsolvable trial, the same container was screwed onto the board so that it was no longer possible to overturn.

### Testing

#### WSC wolves and dogs

Wolves and pack-living dogs were tested in an indoor testing area at the Wolf Science Center, Austria, with an unfamiliar experimenter and a trainer present in the area. In some cases, a camera-person was filming, whilst in others, the video-camera was set up on a tripod and remote controlled. The animal was brought into the testing room on a collar by a trainer. The apparatus was present in the room, unbaited, before the animal was brought in. The animal could initially explore the room and the apparatus for a few minutes before testing began. The trainer and experimenter stood approximately 50 to 75 cm away from adjacent sides of the board. While the trainer held the animal by their side, the experimenter held some food in their hand, showed it to the animal and baited the apparatus by placing the food on the wooden board and covering it with the container. The animal was then released, whilst all people present in the room stood silently avoiding direct eye contact with the animal.

Solvable trials were terminated after 3 minutes or after the animal obtained the food. Only animals that could successfully obtain food in all three solvable trials were tested with the unsolvable apparatus. The unsolvable trial, which also lasted 3 minutes, consisted of the same apparatus but with the container fastened to the wooden board with screws. When a solvable trial ended, the trainer/owner called the animal back and held it by the collar while the experimenter re-baited the apparatus.

#### Pet dogs & Free-ranging dogs

The test procedure was almost identical to that for the Wolf Science Center wolves and dogs apart from a few minor adjustments. First, free-ranging dogs and pet dogs were tested outdoors, on sidewalks or streets and in ‘dog zones’/parks respectively. Second, the experimenter stood between 1 and 1.5 m from the apparatus (a bit further than for wolves and WSC dogs). Third, for pet dogs, the owner was also present during testing and hence adopted the location of the ‘trainer’ in the wolf and dog testing, but for free-ranging dogs, for obvious reasons no owner was present. A camera-person was also always present. After each trial, pet dogs were called back by their owners and held by their collars (just as for wolves and dogs at the WSC) whilst the experimenter re-baited the apparatus. However, in the case of free ranging dogs, to bait the apparatus, the experimenter distracted the animal by tossing a small piece of food a few meters away from the apparatus; hence differently from the animals in the other groups, the exact start location of free-ranging dogs could not be standardized.

As for wolves and WSC dogs, pet and free-ranging dogs needed to be successful in all 3 solvable trials before being presented with the unsolvable trial. Four pet dogs were not able to solve the first solvable trial, so the experimenter moved the container off the board in view of the dogs allowing the dogs to eat the food reward, and then each dog was given 3 more solvable trials (which they then solved).

### Analyses

Following Miklósi *et al*.^3^ and Marshall-Pescini *et al*.^11^ several behaviours were coded from video. The ‘latency to success’ in solvable trials was calculated as the time that passed from the animal first touching the apparatus, to the food being uncovered, which allowed a comparison across groups despite potential differences in the starting location of the animals (see Methods section). In the unsolvable trial: ‘Persistence’ was measured in terms of the duration the animal spent interacting with the apparatus (i.e. pawing, licking, sniffing, scratching, biting, nibbling, pulling and pushing the container or wooden board). ‘Looking back’ (i.e. raising or turning the head and looking towards a human) was coded separately for each person present in the testing area. For the analyses, the latency of looking back consisted of the time that passed from the moment the animal started interacting with the apparatus, to the first look to any person (regardless of identity). The frequency and duration of ‘looking back’ were measured as the sum of the gazes and time spent looking at people present in the test area. In former studies using the unsolvable task paradigm^16^, ‘Gaze alternation’ i.e. looking towards the apparatus immediately followed by a look to the person (or *vice versa*) was suggested as a potentially more stringent measure of communicative behaviours towards human, hence we also included the frequency of occurrence of this behavioural sequence in our analyses. For analyses on the latency, frequency and duration of looking back, we included only animals that had in fact exhibited the behaviour.

Inter-observer reliability was carried out with a second observer coding 20% of the video data (Intra-class correlation coefficient: Gaze human: frequency ICC = 0.9, duration ICC = 0.94, latency ICC = 0.95; Latency to success ICC = 0.99; Duration interact apparatus ICC = 0.99; Frequency Gaze alternation ICC = 0.76). Because the number of people in the test area (experimenter, cameraperson, owner/trainer) was not consistent across dog populations (see procedure above), we ran an generalized linear model (Poisson distribution) to check whether this may have affected the frequency of the dogs’ looking behaviour in the unsolvable trial. We found that the number of individual in the test area had no effect on the frequency of looking back (GLM: χ^2^ = 0.439 p = 0.508).

To assess potential learning effects across solvable trials, a Linear Mixed Model was used, with the latency to success as response variable, trial and group as explanatory factors and the identity of the individuals as the random factor. For the unsolvable trial, linear models were used with either (a) the time spent interacting with the apparatus, (b) latency or (c) duration of looking back as the response variable and group, persistence and the interaction between group and persistence as explanatory factors. Generalized linear models (d) with a binomial distribution for the occurrence of looking back and (e) a quasi-Poisson distribution (to correct for over-dispersion) for the frequency of looking and gaze alternation, were also run with the same explanatory factor. Model reduction based on p-values was carried out. Models (b) to (f) were also run with just group and *not* persistence (i.e. the time spent interacting with the apparatus) as an explanatory variable to allow for comparison with previous studies that had not taken persistence into account. All models were run in the program R (version 3.2), using the package lme4 followed, where necessary, by corrected multiple comparisons using the package multcomp (www.r-project.org).

### Ethical statement

All procedures and methods were discussed and approved by the institutional ethics committee in accordance with Good Scientific Practice guidelines and national legislation and all methods were performed in accordance with the relevant guidelines and regulations. Informed consent was obtained by all owners. Ethical approval for this study was obtained, from the ‘Ethik und Tierschutzkommission’ of the University of Veterinary Medicine (Protocol number ETK-02/02/16 and ETK-03/02/16).

## Additional Information

**How to cite this article:** Marshall-Pescini, S. *et al*. The role of domestication and experience in ‘looking back’ towards humans in an unsolvable task. *Sci. Rep.* **7**, 46636; doi: 10.1038/srep46636 (2017).

**Publisher's note:** Springer Nature remains neutral with regard to jurisdictional claims in published maps and institutional affiliations.

## Supplementary Material

Supplementary Information

Supplementary Dataset

## Figures and Tables

**Figure 1:**
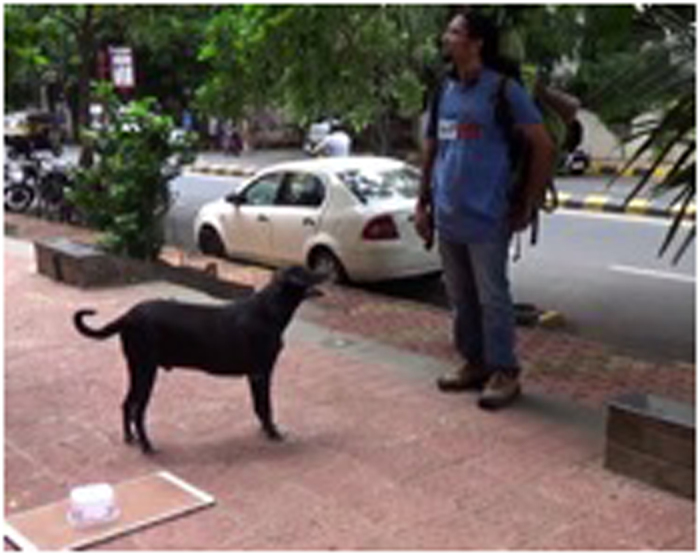
A free-ranging dog on the streets of India ‘looking back’ towards the experimenter during the unsolvable trial.
