# Supplementary material for: The role of domestication and experience in ‘looking back’ towards humans in an unsolvable task
Source: Sci Rep. 2017 Apr 19;7:46636. doi: 10.1038/srep46636 (PMC5395970; doi:10.1038/srep46636)
Supplement: Supplementary Information [file srep46636-s1.doc]

**Supplementary Material**

**“The role of domestication and experience in ‘looking back’ towards humans in an unsolvable task.”**

Sarah Marshall-Pescini, Akshay Rao, Zsófia Virányi, Friederike Range

**Subjects**

***Wolf Science Centre Wolves and dogs***

Differently from previous generations of wolf and dog puppies (see Range and Virányi 2014 for a full description), dog pups raised in 2014 were the offspring of females housed at the WSC. Hence for these animals the raising procedure involved both mother- and human- raising. During the day pups spent their time with human caregivers in a hand-raising enclosure separated from their mother but together with a few of their own littermates and pups from a second litter. During this time, they underwent the same treatment as previous pups raised at the WSC, i.e. copious amount of interaction with both familiar and unfamiliar humans as well as regular bottle-feeding by caregivers. At night, the pups were reunited with their mother and other pack mates in their home enclosures. At 2 months of age pups, 4 pups from each litter were randomly selected to remain at the WSC, whereas the others were given to private owners to be raised as pets. The raising routine described above continued until 5 months of age when, as with previous litters, pups started living on a more permanent basis within their packs. However, they continued to maintain regular and frequent contact with both familiar and unfamiliar humans, and regularly participated in cognitive testing. In all other respects (feeding, medical, testing routines etc.), the raising of the 2014 dog cohort was the same as previous dog and wolf pup litters.

| **Dog/Wolf** | **Name** | **Sex** | **Age at testing (years)** |
| --- | --- | --- | --- |
| **Wolf** | Amarok | M | 3.67 |
|  | Apache | M | 1.09 |
|  | Aragorn | M | 1.4 |
|  | Cherokee | M | 1.09 |
|  | Chitto | M | 3.7 |
|  | Geronimo | M | 1.14 |
|  | Kaspar | M | 1.4 |
|  | Kenai | M | 1.4 |
|  | Nanuk | M | 1.15 |
|  | Shima | F | 1.4 |
|  | Tala | F | 3.7 |
|  | Tatonga | M | 1.17 |
|  | Wamblee | M | 3.62 |
|  | Wapi | M | 1.28 |
|  | Yukon | F | 1.14 |
| **Dogs** | Alika | F | 1.01 |
|  | Banzai* | M | 1.03 |
|  | Enzi* | M | 1.03 |
|  | Gombo* | M | 1.06 |
|  | Hiari* | M | 1.06 |
|  | Imara* | F | 1.06 |
|  | Kilio | M | 0.96 |
|  | Maisha | M | 0.96 |
|  | Nuru | M | 1 |
|  | Panya* | F | 1.03 |
|  | Pepeo* | M | 1.03 |
|  | Rafiki | M | 1 |
|  | Sahibu* | M | 1.06 |
|  | Zuri | F | 1 |

Table S1: subjects housed at the Wolf Science Centre participating in the study. * indicates dogs that were only partly raised as the wolves and the other dogs

***Free-ranging dogs***

Our aim in testing free-ranging dog was to assess how a population with an extremely different relationship with humans would compare to pet dogs in the unsolvable task. The selected population of free-ranging dogs was hence chosen as being representative of a group of dogs that are familiar with humans, since they live in the city and scavenge around human habitations, but have not formed strong bonds with them and do not have the same extent of experience with humans ‘helping’ them when confronted with unobtainable objects/food etc. However, for a valid comparison, it was important for us to test dogs that did not fear humans. Accordingly, a *pre-test* was conducted with free ranging dogs to ascertain they were comfortable enough with the experimenter to conduct the test.

*Pre-test* conducted with free-ranging dogs: Once a potential subject was spotted, the experimenter walked in the direction of the dog without making direct eye contact. From 2 to 3 meters, the experimenter attempted to catch the dog’s attention then tossed a piece of sausage in the direction of the dog and took a few steps away (stage 1). If the dog showed signs of fear or stress (back crouching, tail tucked between legs, walking away from the person, rapidly pacing towards the food and away from it, growling or barking at the experimenter) the experimenter walked away from the dog without any further interaction. If the dog consumed the food, the experimenter then kneeled and placed a piece of sausage about 20 – 30 cm from his feet (stage 2). If the dog was hesitant to approach, the experimenter slowly took a few steps back, away from the dog. A maximum of three food presentations at this distance were carried out. If, after 3 trials, the dog still showed signs of fear or stress, it was excluded from further testing. If the dog approached the experimenter and consumed the food at least once, it moved on to stage 3. In stage 3, the experimenter held a piece of sausage in the palm of his extended arm towards the dog offering the food. If the dog did not approach and consume the food from the hand, the experimenter placed it on the ground just in front of him and remained still. Dogs went on to be tested if they consumed the food with no signs of stress at least in stage 2. Out of the 46 dogs that underwent the pre-test, 9 dogs did not pass stage 1, 4 dogs passed stage 2 and 26 dogs passed stage 3. Seven dogs approached the experimenter without any signs of fear or stress, sniffed the food but did not consume it. These dogs were not included in testing. The experimenter also offered all pet dogs a piece of food from his hand prior to starting the test.

**Results**

| **Latency to success** | **df** | **Chisq** | **p** |
| --- | --- | --- | --- |
| Trial | 1 | 116.83 | <0.0001 |
| Group | 3 | 58.08 | <0.0001 |
| Trial:Group | 3 | 0.93 | 0.82 |

Table S2: Results of the linear mixed model on the latency to success in the solvable trials.

| **Group comparison**  **(latency to success)** | **z** | **p** |
| --- | --- | --- |
| Free-ranging vs. Pets | 2.53 | 0.056 |
| WSC dogs vs. Pets | 0.07 | 0.9 |
| Free-ranging vs. WSC dogs | 2.43 | 0.069 |
| Wolves vs. WSC dogs | 5.02 | <0.001 |
| Wolves vs. Free-ranging | 7.17 | <0.001 |
| Wolves vs. Pets | 5.47 | <0.001 |

Table S3: corrected post-hoc group comparisons for the latency to success in the solvable trials. Mean seconds to success across all three solvable trials: wolves: 4.2; pets: 6.4; free-ranging 19.7; WSC dogs: 14.4.

| **Group comparison (persistence)** | **t** | **p** |
| --- | --- | --- |
| Free-ranging vs. Pets | 1.05 | 0.72 |
| WSC dogs vs. Pets | 0.08 | 0.99 |
| Free-ranging vs. WSC dogs | 0.91 | 0.79 |
| Wolves vs. WSC dogs | 4.32 | <0.001 |
| Wolves vs. Free-ranging | 3.12 | 0.015 |
| Wolves vs. Pets | 4.73 | <0.001 |

Table S4: corrected post-hoc group comparisons of the time spent interacting with the apparatus in the unsolvable trial. Wolves: mean 110, range: 39.4-178.7; WSC dogs mean: 46.6 seconds, range: 32.4-69.6; FRD mean: 60.46 seconds, range: 20.5-110.3; PD mean: 48 seconds, range: 5.8-155.9.

| **Occurrence of looking back** | **df** | **Chisq** | **p** |
| --- | --- | --- | --- |
| Group | 3 | 11.85 | 0.008 |
| Persistence | 1 | 21.7 | <0.0001 |
| Group:Persistence | 3 | 1.8 | 1 |

Table S5: Results of the generalized linear model (binomial) run on the likelihood of ‘looking back’ occurring in the unsolvable trial, in relation to persistence and group.

| **Latency to look back** | **df** | **F** | **p** |
| --- | --- | --- | --- |
| Group | 3 | 1.04 | 0.38 |
| Persistence | 1 | 11.9 | 0.001 |
| Group:Persistence | 3 | 0.67 | 0.57 |

Table S6: Results of the linear model on the latency to look back in the unsolvable trial, considering only those animals that exhibited the behaviour.

| **Duration of looking back** | **df** | **F** | **p** |
| --- | --- | --- | --- |
| Group | 3 | 2.02 | 1.22 |
| Persistence | 1 | 33.41 | 0.001 |
| Group:Persistence | 3 | 0.14 | 0.94 |

Table S7: Results of the linear model on the duration of looking back in the unsolvable trial, considering only those animals that exhibited the behaviour.

| **Frequency of looking back** | **df** | **Chisq** | **p** |
| --- | --- | --- | --- |
| Group | 3 | 5.22 | 0.16 |
| Persistence | 1 | 27.79 | 0.0001 |
| Group:Persistence | 3 | 3.68 | 0.3 |

Table S8: Results of the generalized linear model on the frequency of looking at the person in the unsolvable trial, considering only those animals that exhibited the behaviour.

| **Group comparison**  **(duration of looking back)** | **t** | **p** |
| --- | --- | --- |
| Free-ranging vs. Pets | 0.45 | 0.97 |
| WSC dogs vs. Pets | 0.08 | 0.99 |
| Free-ranging vs. WSC dogs | 0.5 | 0.96 |
| Wolves vs. WSC dogs | 4.19 | 0.001 |
| Wolves vs. Free-ranging | 3.42 | 0.006 |
| Wolves vs. Pets | 4.4 | 0.001 |

Table S9: corrected post-hoc group comparisons of the time spent looking back at the person in the unsolvable trial, including only animals that exhibited this behaviour, following a model which did *not* include persistence as an explanatory factor. Looking back duration for wolves mean: 7.35, range: 0.8-25; WSC-dogs mean: 14, range: 4-28; PD mean: 12 range: 3-25; FRD mean: 13, range: 4-28).

| **Group comparison**  **(frequency looking back)** | **z** | **p** |
| --- | --- | --- |
| Free-ranging vs. Pets | 0.62 | 0.92 |
| WSC dogs vs. Pets | 0.59 | 0.93 |
| Free-ranging vs. WSC dogs | 0.07 | 0.99 |
| Wolves vs. WSC dogs | 3.33 | 0.005 |
| Wolves vs. Free-ranging | 3.26 | 0.006 |
| Wolves vs. Pets | 2.97 | 0.015 |

Table S10: corrected post-hoc group comparisons for the frequency of looking back at the person in the unsolvable trial, including only animals that exhibited this behaviour, following a model which did *not* include persistence as an explanatory factor. Looking back frequency for wolves mean: 73, range: 1-25; WSC-dogs mean: 14, range: 4-28; PD mean: 12 range: 3-25; FRD mean: 13, range: 4-28).

| **Frequency of gaze alternation** | **df** | **Chisq** | **p** |
| --- | --- | --- | --- |
| Group | 3 | 8.74 | 0.033 |
| Persistence | 1 | 11.39 | 0.0007 |
| Group:Persistence | 3 | 0.54 | 0.91 |

Table S11: Results of the generalized linear model on the frequency of gaze alternations in the unsolvable trial.

| **Group comparison**  **(frequency gaze alternations)** | **z** | **p** |
| --- | --- | --- |
| Free-ranging vs. Pets | 2.07 | 0.15 |
| WSC dogs vs. Pets | 2.55 | 0.05 |
| Free-ranging vs. WSC dogs | 0.23 | 0.99 |
| Wolves vs. WSC dogs | 1.48 | 0.44 |
| Wolves vs. Free-ranging | 1.27 | 0.57 |
| Wolves vs. Pets | 0.31 | 0.99 |

Table S12: corrected post-hoc group comparisons for the frequency of gaze alternation between person and apparatus (or vice versa) in the unsolvable trial (including all animals). Average frequency of gaze alternations for wolves = 1.8 (range: 0-7) WSC dogs= 7.3 (range: 1- 20), PD=2.9 (range: 1-17) and FRD=6 (range: 1-14).

| **Duration interact person** | **df** | **F** | **p** |
| --- | --- | --- | --- |
| Group | 3 | 1.7 | 0.19 |
| Persistence | 1 | 7.69 | 0.007 |
| Group:Persistence | 3 | 0.83 | 0.48 |

Table S13: Results of the generalized linear model on the duration of human interaction.
